# Expression of protein disulfide isomerase family members correlates with tumor progression and patient survival in ovarian cancer

**DOI:** 10.18632/oncotarget.21569

**Published:** 2017-10-06

**Authors:** Soma Samanta, Shuzo Tamura, Louis Dubeau, Paulette Mhawech-Fauceglia, Yohei Miyagi, Hisamori Kato, Rich Lieberman, Ronald J. Buckanovich, Yvonne G. Lin, Nouri Neamati

**Affiliations:** ^1^ Department of Medicinal Chemistry, College of Pharmacy, University of Michigan, Ann Arbor, Michigan; ^2^ USC/Norris Comprehensive Cancer Center, Keck School of Medicine, University of Southern California, Los Angeles, CA, USA; ^3^ Kanagawa Cancer Center Research Institute, Yokohama, Japan; ^4^ Department of Internal Medicine, Division of Hematology Oncology, Division of Gynecologic Oncology, University of Michigan, Ann Arbor, Michigan; ^5^ Current/Present affiliation: Magee-Womens Research Institute, University of Pittsburgh, Pittsburgh, PA, USA; ^6^ Current/Present affiliation: Genentech-Roche, South San Francisco, California, USA

**Keywords:** tissue microarray (TMA), ovarian cancer, PDI family proteins, clinical outcome, patient survival

## Abstract

**Objective:**

Protein disulfide isomerase (PDI) is an oxidoreductase that is overexpressed in several cancers. PDI family members (PDIs) play a role in various diseases including cancer. Select PDIs were reported as useful markers in other cancers but their expression in ovarian cancer has not been thoroughly assessed. We sought to evaluate the expression of PDI, PDIA6, PDIR, ERp57, ERp72 and AGR3 in ovarian cancer patient samples and examine their prognostic significance.

**Methods:**

TMA samples from 415 tissues collected from three cancer centers (UM, USC, and KCCRI) were used to assess the expression levels of PDI family proteins using IHC.

**Results:**

We observed significant increases in PDI (*p* = 9.16E-36), PDIA6 (*p* = 5.51E-33), PDIR (*p* = 1.81E-12), ERp57 (*p* = 9.13E-07), ERp72 (*p* = 3.65E-22), and AGR3 (*p* = 4.56E-24) expression in ovarian cancers compared to normal tissues. Expression of PDI family members also increases during disease progression (*p* <0.001). All PDI family members are overexpressed in serous ovarian cancer (*p*<0.001). However, PDI, PDIA6, PDIR, ERp72 and AGR3 are more significantly overexpressed (*p*<0.001) than ERp57 (*p*<0.05) in clear cell ovarian carcinoma. Importantly, overexpression of PDI family members is associated with poor survival in ovarian cancer (*p* = 0.045 for PDI, *p* = 0.047 for PDIR, *p* = 0.037 for ERp57, *p* = 0.046 for ERp72, *p* = 0.040 for AGR3) with the exception of PDIA6 (*p* = 0.381).

**Conclusions:**

Our findings demonstrate that select PDI family members (PDI, PDIR, ERp72, ERp57 and AGR3) are potential prognostic markers for ovarian cancer.

## INTRODUCTION

Epithelial ovarian cancer (EOC) is the fifth most common cause of cancer death among women [1, 2]. Although death rates from EOC have been decreasing on an average 2.0% each year from 2003 to 2013 due to advances in diagnosis and treatment, mortality rates are still high,[3–5] displaying the 3^rd^ highest incidence to mortality ratio among all cancers. On the basis of histopathology and molecular genetics, EOCs are divided into five types: high-grade serous (70%), endometrioid (10%), clear cell (10%), mucinous (3%) and low-grade serous carcinomas (<5%) [6, 7]. Recent studies showed that EOCs are genetically heterogeneous even within individual subtypes [8, 9]. Drug resistance is the major cause of treatment failure resulting in death of >90% of patients with metastatic disease [10]. In contrast, approximately 95% of patients live longer than 5 years after diagnosis when ovarian cancer is detected early and the disease is localized. Therefore, identification of effective biomarkers for early stage diagnosis is needed to improve survival rates.

Previously, we discovered small-molecule propionic acid carbamoyl methyl amides (PACMAs) targeting protein disulfide isomerase (PDI) that elicited anticancer activity in EOC models [11]. PDI belongs to a superfamily of oxidoreductase proteins residing in the endoplasmic reticulum (ER), nucleus, cytosol, mitochondria and cell membrane [12, 13] whose function is to maintain cellular homeostasis. In addition, it is also known for its oncogenic and pro-survival functions in different cancer types [14, 15]. Consistent with this notion, PDI *(P4HB)* is overexpressed in a variety of cancer types including brain, lymphoma, kidney, prostate, lung and ovarian cancers (Supplementary Figure 1). PDI overexpression in several cancers correlates with poor clinical outcome and increased metastasis, invasion and chemoresistance [15, 16].

Select PDI family members were reported to play important roles in cancer progression and chemoresistance through undefined mechanisms. For example, ERp57 (*PDIA3*), plays a role in paclitaxel-resistance [17, 18] and Anterior Gradient 3 (*AGR3*) mediates cisplatin resistance in EOC [19]. ERp72 (*PDIA4)* and ERp5 (*PDIA6)* mediate resistance to cisplatin-induced cell death in lung adenocarcinoma [20]. PDIR (*PDIA5*) activates the process of ATF6-alpha-packaging into coat protein complex II vesicles and is required for the development of chemoresistance [21]. Regarding cancer progression, *PDIA3* and *PDIA6* gene expression are established markers of aggressiveness in primary ductal breast cancer [22]. PDIA6 also promotes proliferation of HeLa cells [23] and its downregulation inhibits proliferation and invasion in bladder cancer [24]. The thiol oxidoreductase ERp57 is an ER resident protein that plays an important role in disulfide bond formation. Knockdown of ERp57 enhances the apoptotic response to anticancer treatment in HCT116 colon cancer cells [25]. Another family member, ERp19 contributes to tumorigenicity in human gastric cancer by promoting cell growth, migration and invasion [26]. PDI family members are consistently elevated in mammospheres suggesting that activation of PDI family proteins during anchorage-independent growth of breast cancer cells plays important and non-redundant roles in their anchorage-independent cell proliferation [27].

Collectively, these data suggest that several PDI family members are associated with disease progression and chemoresistance in several cancers, but little is known about their role in EOC. Using TMA samples, we demonstrated that PDI, PDIA6, PDIR, ERp57, ERp72 and AGR3 are overexpressed in EOC tumors. More importantly, PDIs overexpression in serous ovarian carcinoma correlates with patient survival, suggesting that PDI, PDIR, ERp57, ERp72 and AGR3 are potential prognostic markers for EOC.

## RESULTS

### Patient characteristics

Patient characteristics are summarized in Table 1. A total of 415 ovarian tumor tissue samples were collected from patients: 89 cases from USC, 192 cases from UM and 134 cases from KCCRI. The majority of patients were diagnosed with tumors of serous histology, late stage, and high histological grades.

**Table 1:** Clinico-pathological characteristics of the patients with ovarian tumors

Patient population	USC		UM		KCCRI	
**Patients’ number**	89		192		134	
**Tumor type**	Serous	35(39.3%)	Serous	152(79.2%)	Serous	51(38.1%)
	Endometrioid	17(19.1%)	Endometrioid	1(0.5%)	Endometrioid	18(13.4%)
	Clear Cell	10(11.2%)	Clear Cell	3(1.6%)	Clear Cell	38(28.4%)
	Mucinous	17(19.1%)	Mucinous	0(0%)	Mucinous	15(11.2%)
	Rare subtype or low grade ovarian cancer	5(5.6%)	Rare subtype or low grade ovarian cancer	2(1.0%)	Rare subtype or low grade ovarian cancer	2(1.5%)
	Other	5(5.6%)	Other	10(5.2%)	other	9(6.7%)
			Mixed ovarian cancer^*^	24(12.5%)		
**Stage**	Benign	8(9.0%)	Benign	18(9.5%)	Benign	1(0.7%)
	Stage I	36(40.4%)	Stage I	9(4.8%)	Stage I	56(41.8%)
	Stage II	6(6.7%)	Stage II	17(9%)	Stage II	16(11.9%)
	Stage III	31(34.8%)	Stage III	112(59.3%)	Stage III	40(29.9%)
	Stage IV	8(9.0%)	Stage IV	30(15.9%)	Stage IV	16(11.9%)
			Not known^**^	3(1.6%)	Not known^**^	5(3.7%)
**Grade**	Grade 0	1(1.1%)	Benign	18(9.5%)		
	LMP	13(14.6%)	Grade 1	19(10.1%)		
	Benign	8(9.0%)	Grade 2	4(2.2%)		
	Grade 1	19(21.3%)	Grade 3	134(70.9%)		
	Grade 2	6(6.7%)	Not known^**^	14(7.4%)		
	Grade 3	42(47.1%)				
**Type of Chemotherapy**	No chemotherapy	31(34.8%)			No chemotherapy	6(4.5%)
	Platinum containing	5(5.6%)			Platinum containing	3(2.2%)
	Platinum and taxol containing	53(59.5%)			Platinum and taxol containing	107(79.9%)
					No Information	18(13.4%)
**Race**	Asian	20(22.5%)				
	Black	2(2.2%)				
	Native American	3(3.3%)				
	White	64(71.9%)				

^*^Mixed ovarian cancer (serous-endometrioids/serous-clear cell carcinoma/ endometrioids-clear cell carcinoma);^**^Not known (Either it was not clinically determinable or the data was not available).

### USC population

Distribution of histologic subtypes seen in the USC cohort was as follow: serous ovarian cancer (39.9%), endometrioid (19.1%), clear cell ovarian carcinoma (11.2%), mucinous ovarian cancer (19.1%), low grade serous ovarian cancer (5.6%), and other ovary related cancers (5.6%). Histological grade consisted of 1.1% grade 0, 14.6% LMP (low malignant potential), 9.0% benign tumor, 21.3% grade 1 tumor, 6.7% grade 2 tumor, 47.1% grade 3 tumor. The population was comprised of 40.4% Stage I, 6.7% Stage II, 34.8% Stage III, 9.0% Stage IV. Out of 89 patients, 31 (34.8%) did not receive chemotherapy, including 27 diagnosed with early stage disease and four that were unfit or declined to receive chemotherapy. The cohort was composed of 22.5% Asian, 2.2% Black, 3.3% Native American, and 71.9% Caucasian patients.

### UM population

The UM cohort was composed of 9.5% benign ovarian lesions (including serous cystadenoma, adenofibroma, endometriotic cysts, etc.) and 94.8% malignant samples. Malignant samples include 79.2% serous, 0.5% endometrioid, 1.6% clear cell, 1.0% low grade ovarian cancer, and 12.5% of cancers with mixed or other histologies. Histological grades were 9.5% benign, 10.1% grade 1, 2.2% grade 2, 70.9% grade 3, 7.4% unknown. The malignant tumors were 4.8% stage I, 9.0% stage II, 59.3% stage III, 15.9% stage IV, and 1.6% of unknown stage. For patients with malignant disease, 97.5% received at least one dose of adjuvant platinum/taxane based chemotherapy. 9.4% were treated neoadjuvantly. Data from tumors for which tumor stage or grade were unknown were censored for overall survival analysis.

### KCCRI population

The KCCRI population was comprised of 38.1% serous, 13.4% endometrioid, 28.4% clear cell, 11.2% mucinous, 1.5% rare or low grade ovarian cancers while 6.7% had other ovarian related histologies. Regarding the FIGO stage, the study group encompassed 0.7% benign, 41.8% stage I, 11.9% stage II, 29.9% stage III, 11.9% stage IV and 3.7% unknown stage. Out of 134, only 6 (3.1%) patients did not receive chemotherapy, 82.1% patients received platinum based chemotherapy, and for 13.4% patients' chemotherapy information was unknown.

### Expression of PDIs in ovarian cancer cell lines and xenografts

**Figure 1:**
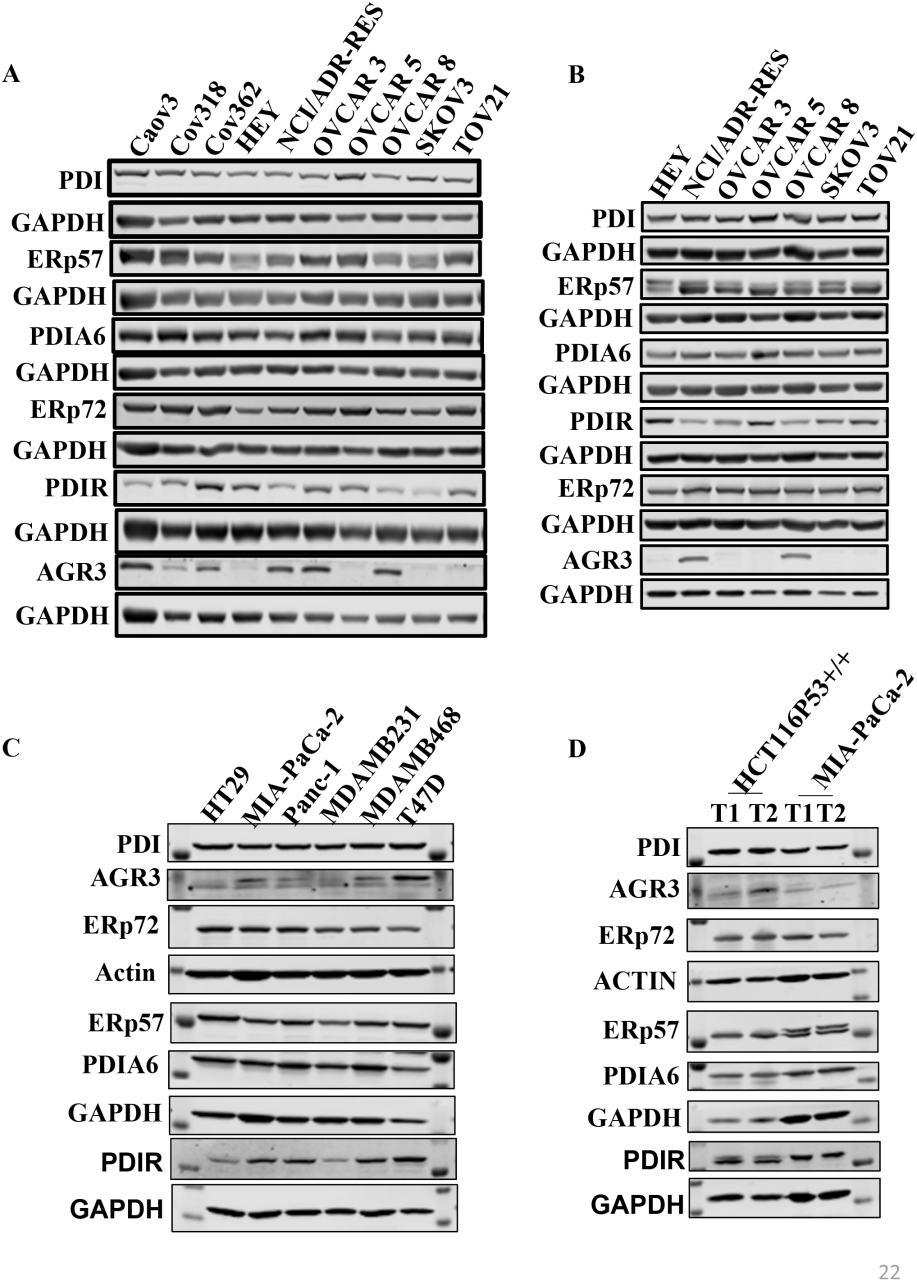
PDI family members (PDIs) are highly expressed in ovarian cancer cells and xenograft **(A)** Expression levels of PDIs in a panel of ovarian cancer cell lines (1 of 3 representative experiments is shown) **(B)** Expression level of PDIs in a panel of ovarian cancer xenografts. **(C)** Expression levels of PDIs in a panel of non-ovarian cancer cell lines (1 of 3 representative experiments is shown). **(D)** Expression levels of PDIs in non-ovarian cancer tissue collected from xenograft.

Initially, we determined the expression of PDI, PDIA6, PDIR, ERp57, ERp72, and AGR3 in ten OC cell lines by Western blotting (Figure 1A, Supplementary Figure 2). PDI, PDIA6, ERp57, and ERp72 were highly expressed in almost all cell lines. PDIR had moderate expression in all cell lines and was higher in Caov3, COV 362, NCI/ADR-RES, OVCAR 3, OVCAR 8 cells. AGR3 was expressed in only half of the cell-lines.

We also determined the expression of PDI proteins in various EOC mouse xenografts. Collected tumor tissues from each xenograft were analyzed by Western blot. PDI, PDIA6, ERp57, ERp72 showed strong expression in all xenograft samples (Figure 1B). HEY, OVCAR5, SKOV3, and TOV21G xenografts had higher PDIR expression than NCI/ADR-RES, OVCAR3 and OVCAR8 xenografts. AGR3 was only expressed in NCI/ADR-RES and OVCAR8 xenografts. All proteins under consideration were highly expressed by IHC and their expression levels correlated with Western blot results, except for AGR3.

We determined the expression of PDI proteins in a panel of non-ovarian cancer cells lines from colon (HT29), pancreatic (MIA PaCa-2 and Panc-1) and breast (MDA-MB-231, MDA-MB-468 and T47D) (Figure 1C, Supplementary Figure 3) for comparison to ovarian cancer cells. Western blot analysis revealed that PDI expression is low in pancreatic cancer cells compared to other cell lines; MIA PaCa-2 cells possessed the lowest expression of PDI, ERp57, PDIA6, and AGR3. Breast cancer cells showed moderate to high expression of most PDI family proteins. T47D cells showed high expression of PDI, ERP57, AGR3, and PDIA6 but not ERp72. ERp57 expression is low in MDA-MB-231 and MDA-MB-468 cell lines. All PDI family proteins were highly expressed in the colon cancer cell line HT29, except for PDIR. Analysis of published data from tumor samples using Oncomine™ [28] also supports these findings (Supplementary Figure 1).

We also determined the protein expressions in non-ovarian xenograft tissues (Figure 1D, Supplementary Figure 4 colon cancer (HCT116) and pancreatic cancer (MIA PaCa-2) xenografts). PDI family proteins expression is low in pancreatic cancer xenografts but high in colon cancer xenografts.

### Distribution of PDI family protein expression in ovarian cancers

**Figure 2:**
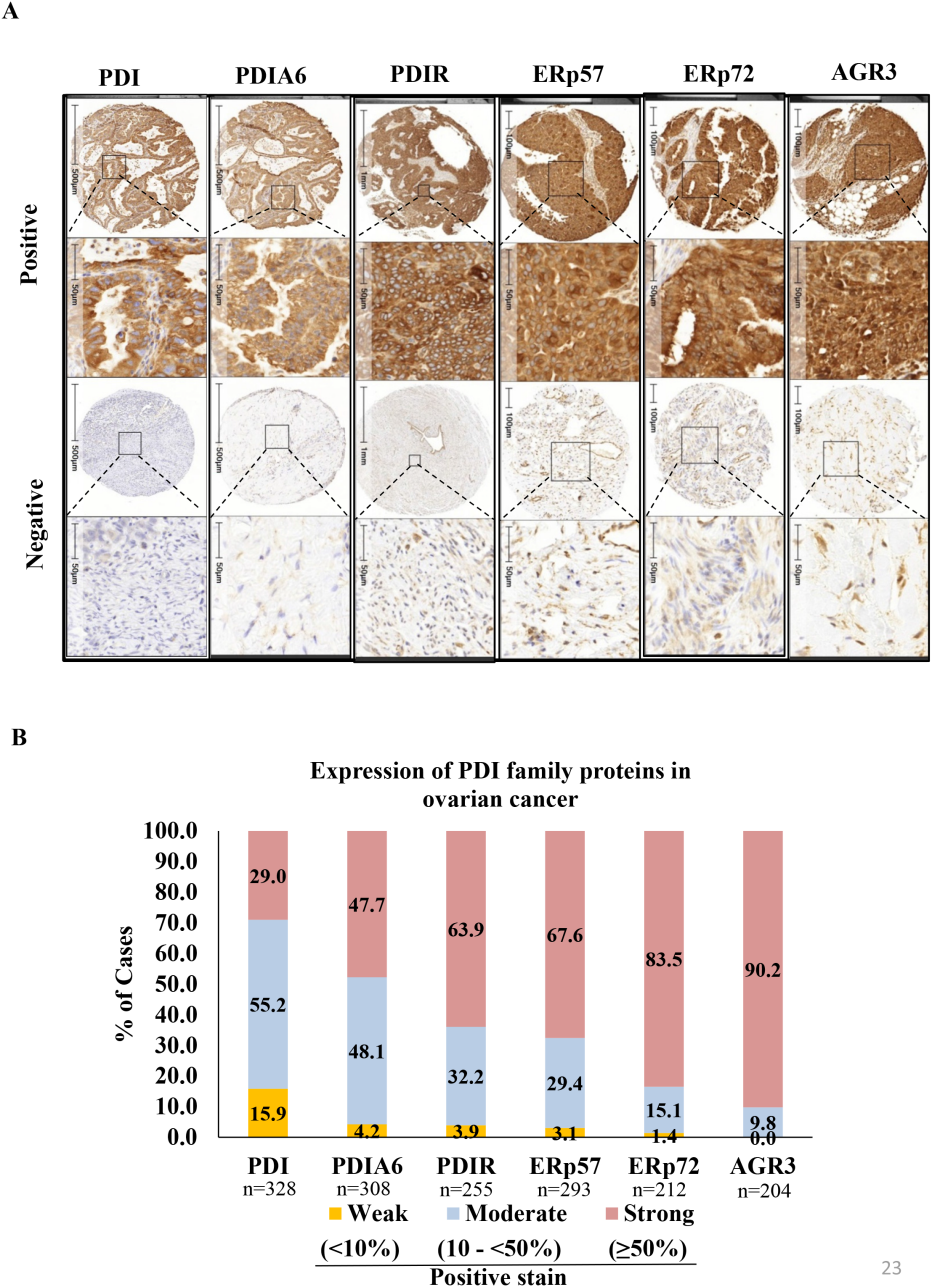
Immunohistochemical expression patterns of PDIs in ovarian cancer tissue samples **(A)** Representative examples of positive and negative/weakly positive staining pattern of PDI, PDI family protein in ovarian cancer tissues **(B)** PDI family protein expression in ovarian cancers. % of staining automatically quantified by HALO (Indica labs informed pathology). % of cases of strong, moderate and weak expression for each protein was plotted in stacked columns in Microsoft Excel.

Expression of PDI family proteins was evaluated in all TMAs. Strong, moderate and weak staining was defined as positivity in ≥50%, 10 to <50% and <10% of the tumor cell population, respectively (Figure 2B). Over 50% of patient samples showed ‘strong’ expression for PDIR, ERp57, ERp72 and AGR3. Representative staining patterns (positive and negative/weakly stained) of proteins are depicted in Figure 2A. Among the 328 ovarian carcinomas stained for PDI, 95 (29%) showed strong, 181 (55.2%) moderate, and 52 (15.9%) weak staining patterns. For PDIA6, among 308 ovarian carcinomas, 147 (47.7%) tissues expressed strong, 148 (48.1%) moderate, and 13 (4.2%) weak positive staining. For PDIR staining of 255 ovarian carcinomas, 163 (63.9%) showed strong, 82 (32.2%) moderate and 10 (3.9%) weak tissue staining patterns. Out of 293 ERp57-stained OC tissues, 198 (67.6%) showed strong, 86 (29.4%) moderate, and 9 (3.1%) weak positivity. Out of 212 ERp72 stained OC tissues, 177 (83.5%) showed strong, 32 (15.1%) moderate, and 3 (1.4%) weak positivity. Out of 204 AGR3 stained OC tissues, 184 (90.2%) showed strong, 20 (9.8%) moderate, and none (0%) weak positivity.

### Expression of PDIs is correlated with patients’ pathological parameters

**Figure 3:**
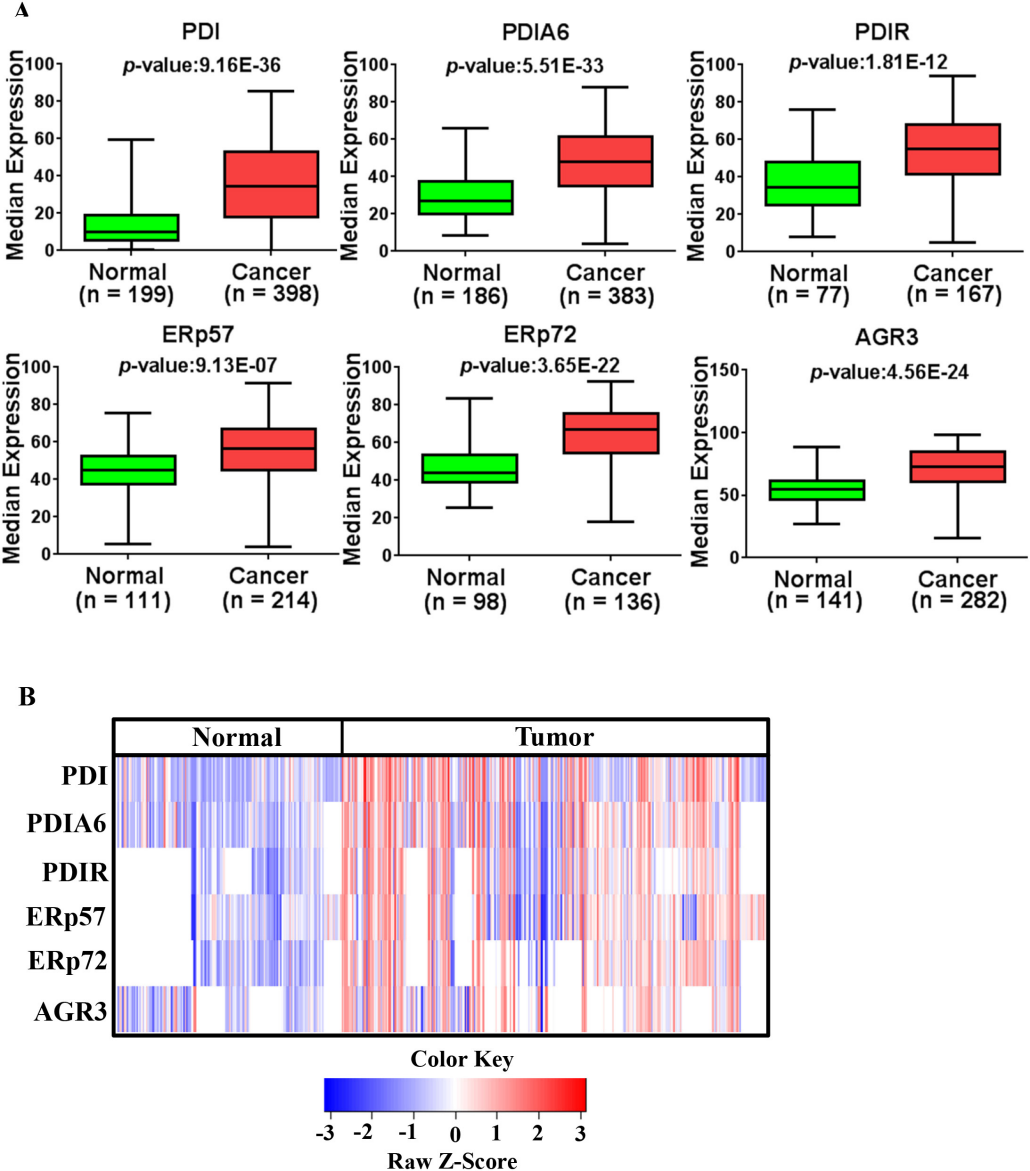
Expression of PDI family members (PDIs) in ovarian cancer **(A)** PDI family members were highly expressed in ovarian cancer with respect to normal tissues. Data sets used for the analysis were obtained from ovarian cancer patients in US (USC and UM) and Japan (KCCRI) and analyzed using Prism 6.0 (GraphPad Software, Inc). **(B)** Expression patterns of PDI family proteins are represented as a heat map generated in R-studio.

The majority of EOC patient samples had strong expression of PDI proteins except for PDI and PDIA6 (29.0% and 47.7% strong expression respectively). Next, we compared the expression in cancerous tissues to that in normal tissues (normal tissue refers to non-tumor control; see Materials and Methods Section). PDI proteins are highly expressed in EOC compared to normal tissues (Figure 3A, *p* = 9.16E-36 for PDI, *p* = 5.51E-33 for PDIA6, *p* = 1.81E-12 for PDIR, *p* = 9.13E-07 for ERp57, *p* = 3.65E-22 for ERp72, *p* = 4.56E-24 for AGR3). Expression patterns of PDI proteins are displayed in a heatmap in Figure 3B and expression patterns in fold change are presented in Supplementary Figure 5. It has been reported that upregulation of PDIs occurs at the mRNA and protein level in many cancers [27, 29]. Similarly, we observed upregulation of PDIs at the mRNA level (Supplementary Figure 6) as well as at the protein level in ovarian cancer.

**Figure 4:**
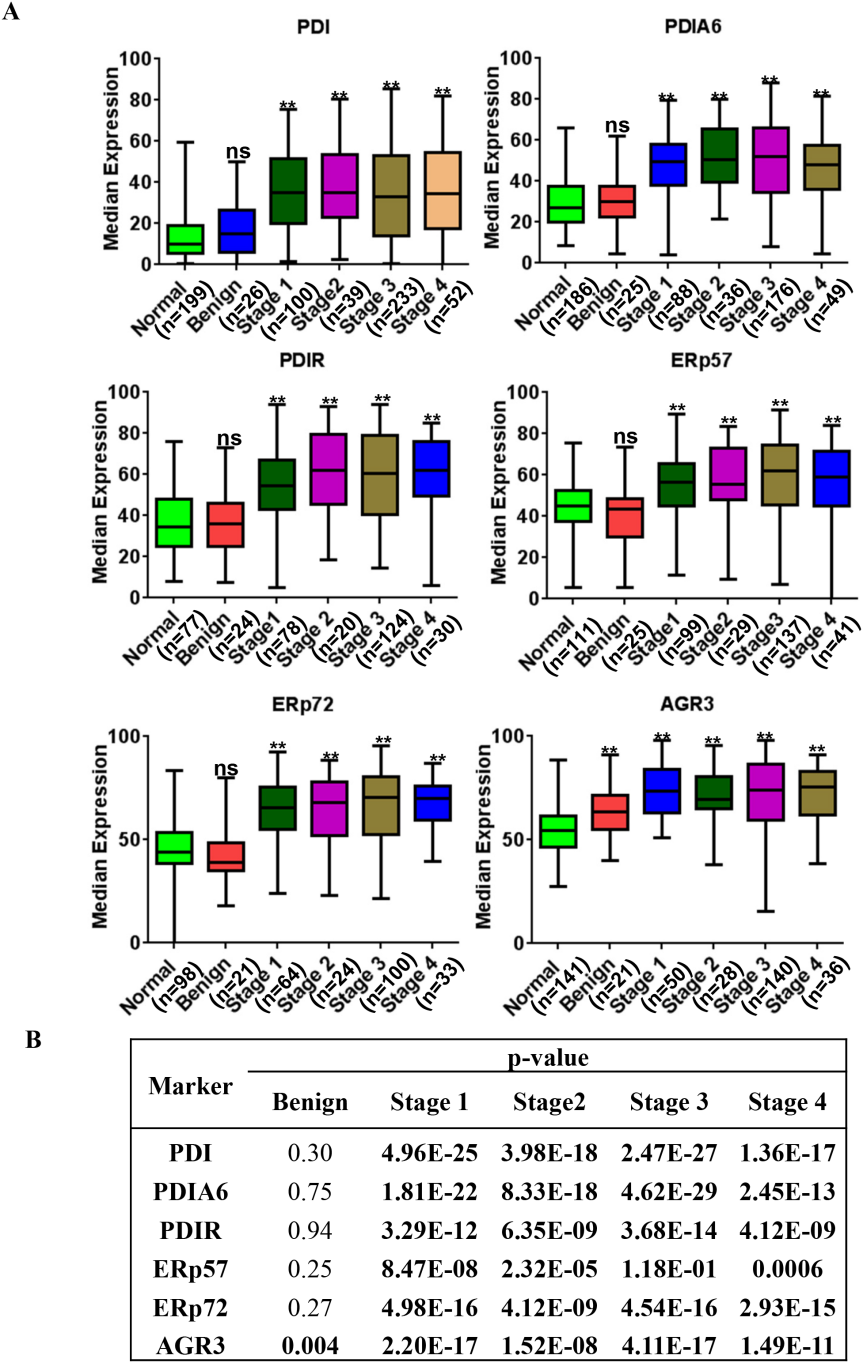
PDI family proteins are overexpressed in tumor tissue collected from tumors of different stages **(A)** Expression of PDI family proteins in normal, benign and malignant tumor tissues from ovarian cancer patients with different stages. **(B)** All PDI family proteins were highly expressed in stage 1 to 4 tissues and were extremely significant when compared to normal, the p values shown in bold characters are statistically significant.^*^p-value <0.05, ^**^p-value <0.005.

We further investigated whether there is a correlation between expression of PDI proteins and clinico-pathological status (Figure 4). Expression of PDI, PDIA6, PDIR, ERp57, ERP72 and AGR3 is significantly correlated with patients’ tumor stages (stage I, stage II, stage III, stage IV, *p* = <0.001). Benign tumor expression patterns were not significantly different than those in normal tissues, except for AGR3, which showed significantly increased expression in benign tumors compared to normal tissue (*p* = <0.005, Figure 4B)

### PDIs are highly expressed in serous ovarian carcinoma

**Figure 5:**
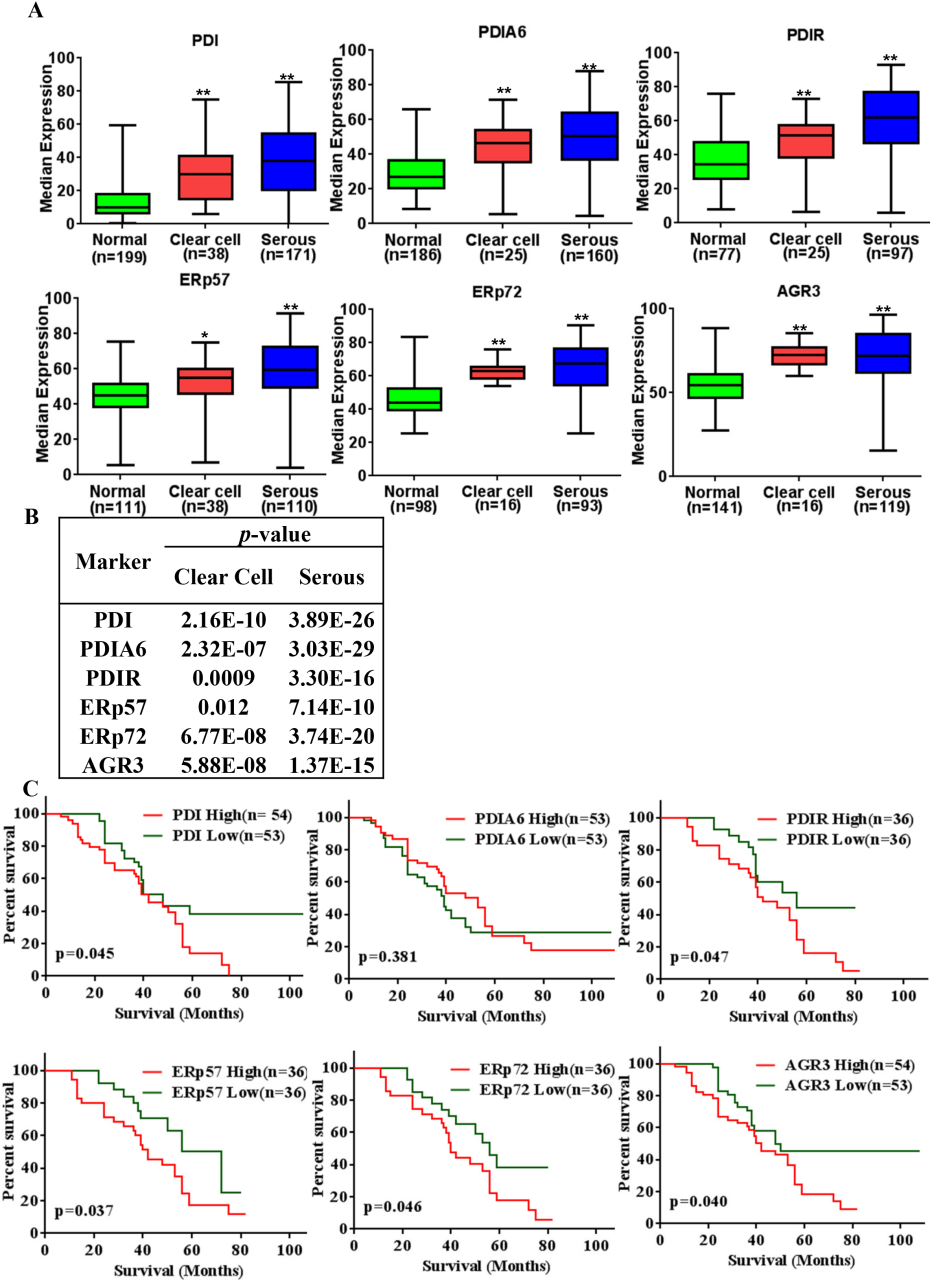
PDI family proteins (PDIs) overexpressed in serous ovarian cancer and expression levels significantly associated with poor overall survival of ovarian cancer **(A)** Expression of PDIs in clear cell ovarian carcinoma and serous ovarian carcinoma. ^*^p-value<0.05, ^**^p-value<0.005 **(B)** p-values for expression compared to the normal, bolds values are statistically significant. **(C)** PDIs expression associated with overall survival rate of the all types of ovarian cancer patients. Data sets were obtained from UM population and analyzed with Prism 6 (GraphPad Software, Inc.). The Kaplan-Meier survival analysis method was used to generate survival curve based on OS data using low (<median expression) and high expression (>median expression) groups.

Next, we evaluated the expression patterns in clear cell and serous ovarian cancer tissues because the majority of the EOC patients showing these histologies were reported as chemotherapy resistant. PDI, PDIA6, PDIR, ERp57, ERp72 and AGR3 were highly expressed in serous ovarian cancer compared to normal tissues (*p* = <0.001) (Figure 5A). Representative staining patterns are shown in Supplementary Figure 7. Expression analysis in clear cell ovarian cancer tissues revealed that PDI, PDIA6, PDIR, ERp72 and AGR3 were likewise highly expressed compared to normal (*p* = <0.001), whereas ERp57 expression is statistically less significant (*p* = <0.05) compared to the above mentioned five proteins in cancerous versus normal tissues (Figure 5B).

### Higher expression of PDIs is correlated with poor patient survival

To evaluate the prognostic significance of PDI and its select family proteins expression, Kaplan-Meier analysis of overall survival (OS) was performed on UM patients (survival data for USC and KCCRI were not available). As shown in Figure 5C the high PDI expression group had a significantly poorer OS than the low PDI expression group (*p* = 0.045). A similar outcome was observed for other family members PDIR (*p* = 0.047), ERp57 (*p* = 0.037), ERp72 (*p* = 0.046) and AGR3 (*p* = 0.040). We did not observe any significant difference between high and low PDIA6 expression curves.

## DISCUSSION

Given the heterogeneity of EOC and the concerns regarding disease relapse due to drug resistance, significant efforts are currently being deployed to identify biomarkers with prognostic value. Current data suggests that PDI is an important drug target for cancer therapy because i) it is highly expressed in several cancers as confirmed by gene expression microarrays and proteome analysis, ii) upregulation of PDI correlates with metastasis, invasion, and migration, iii) PDI plays a role in chemoresistance, and iv) PDI supports tumor survival and cancer progression [15, 30]. Select PDI family members are also involved in a variety of human diseases and disorders like infection and immunity, infertility, lipid homeostasis, hemostasis, neurodegeneration and cancer [12, 31, 32]. Thus, select PDI family members have the potential to be exploited as cancer biomarkers. However, little is known about the correlation between PDIs expression and their prognostic significance in EOC. In this study, we focused on six PDI family proteins: PDIA6, PDIR, ERp57, ERp72, AGR3 and PDI, because these proteins have been found to promote tumor cell proliferation [33, 34] or have unknown cellular functions [35]. AGR3 was chosen because it is poorly understood as a tumor-signaling molecule [36]. In this context, it is relevant to note that posttranslational modification of PDI family proteins (e.g. phosphorylation, glycosylation, nitrosylation, and glutathionylation) may regulate the function of these proteins, however their role in disease progression are not fully established [13, 37–39]. Additionally, there are several transcription factors (SP1, NF-YA, XBP-1)[40–42] that regulate PDI expression, but the mechanism behind their regulation is not well studied in cancer. Among these transcription factors, SP1 is overexpressed in ovarian cancer, while there are no prior studies on the expression of NF-YA and XBP-1 in ovarian cancer (Oncomine™ database [28]). Here, we conducted a methodical evaluation of the expression of PDI and its family members in EOC samples from a diverse patient population. Earlier studies reported that overexpression of PDI may serve as a diagnostic marker for glial cell cancer [16], colorectal cancer [43], hepatocellular carcinoma [44] and breast cancer [45]. We demonstrated that expression of PDIs increased in EOC tissue compared to normal tissue, suggesting that these proteins may serve as diagnostic markers for EOC. PDI, PDIA6, PDIR, ERp57, and ERp72 are highly expressed in ten EOC cell lines and AGR3 is expressed in some cell lines. Results of Western blot analysis on EOC mouse xenografts also revealed that PDI, PDIA6, ERp57, ERp72 are strongly expressed in all xenografts. PDIR expression was higher in xenografts of HEY, OVCAR5, SKOV3, and TOV21G cells than from NCI/ADR-RES, OVCAR3 and OVCAR8 cells. AGR3 was expressed in NCI/ADR-RES and OVCAR8 xenografts only. More significantly, overexpression of PDIs correlated with disease progression, as PDI expression increased significantly with higher tumor stages (stage 1 to stage 4, *p* = <0.001) compared to normal.

Clear cell ovarian carcinomas have lower rates of survival when compared to the more common serous subtype [46]. No prognostic or predictive marker is currently available for this EOC subtype [46, 47]. In clear cell ovarian carcinoma PDI, PDIA6, PDIR, ERp72, ERp57 and AGR3 were overexpressed. To our knowledge, no previous studies reported on the upregulation of PDIs in ovarian clear cell carcinoma. Our results strongly suggest that PDI, PDIA6, PDIR, ERp57, ERp72 and AGR3 expression can be useful biomarkers for clear cell ovarian carcinoma but additional studies are required to further validate the prognostic role of PDIs in these cancers.

Our study revealed that high expression of PDI and its family members are correlated with poor patient survival. This is a first report showing that high levels of PDI, PDIR, ERp57, and ERp72 expression are associated with poor patient’s survival in EOC. For AGR3, we observed that high expression adversely affects OS of ovarian cancer patients. This is in disagreement with previous findings by King *et al.* [48], who reported that higher AGR3 expression is associated with longer median survival in low-grade and high-grade serous ovarian carcinoma. Interestingly, Garczyk *et al.* [49] reported that AGR3 has a prognostic impact on early detection of breast cancer, and that low- and intermediate-grade tumor patients with high AGR3 expression had an unfavorable outcome [49]. Obacz *et al.* reported that AGR3 positivity was associated with better outcome (progression free survival and overall survival) in the subgroup of patients with tumors characterized by lower histological grade but not by higher histological grade in breast cancer [50]. Though AGR3 levels are increased in various cancers, the role of AGR3 in tumors, particularly in ovarian tumors, is poorly understood. To understand the clinicopathological significance or clinical outcome related to AGR3 expression, further studies are required.

In conclusion, our data demonstrate that upregulation of select PDI family members can be considered as risk factors for ovarian cancer patients and PDI, PDIR, ERp57, ERp72 and AGR3 could be used as prognostic biomarkers. Because these biomarkers are highly expressed in Stage I disease their expression should be further assessed in plasma and circulating tumor cells for early diagnosis.

## MATERIALS AND METHODS

### Patients and study population

Tissue specimens from three distinct patient populations were collected at USC Norris Cancer Center (Los Angeles, CA), UM Comprehensive Cancer Center (Ann Arbor, Michigan), and KCCRI (Kanagawa Cancer Center Research Institute, Yokohama, Japan). Clinical information and follow-up data were obtained from medical records. Informed consent was obtained from all patients prior to tissue procurement. All studies were performed with the approval of the Institutional Review Board of the respective center. The main clinical and pathological variables evaluated in this study are shown in Table 1. All patients were staged according to the FIGO classification and tumors were graded according to the World Health Organization (WHO) criteria. All non-tumor and tumor tissues used in this study were confirmed by histopathologists from the respective centers.

### Non-tumor control

The term ‘normal’ used in this article was defined as non-tumor tissues collected from normal ovaries at the time of removal of a paratubal cyst or follicular cyst and from patients with benign adenofibromas, serous cystadenomas, endometriosis, and/or mucinous cystadenomas (UM). Because of the recent consensus on the origin of the EOC, non-tumor tissues were also collected from fallopian tubes, especially from the fimbriae. During normal tissue collections, the corpus albicans was excluded (KCCRI). Representative staining of the PDI family proteins in non-tumor tissues are depicted in Supplementary Figure 8. We pooled all the normal tissues collected from UM and KCCRI and compared the PDIs expression against tumor tissues collected from all three patient populations (USC, UM and KCCRI). Two hundred non-tumor tissues samples were used as control in this study.

### TMA

Formalin–fixed and paraffin-embedded tumor and non-tumor tissues from EOC patient samples were used for TMA construction. After review and confirmation by the histopathologist from each center, a tissue microarray was constructed as described previously by Kononen *et al.* [51]. Briefly, after carefully choosing the morphologically representative region from the hematoxylin-eosin section, either 1 mm, 2 mm, or 4 μm cores (for samples collected from USC, KCCRI, or UM respectively) were punched from the selected paraffin-embedded donor blocks and transferred to the paraffin-embedded receiver block. To overcome tumor heterogeneity, core biopsies were performed from two/three different areas of each tumor and tissues were spotted in duplicates/triplicates on the TMA slides.

### Immunohistochemistry and automated analysis

Immunohistochemical (IHC) staining was performed at the Pathology Core of the University of Michigan. IHC staining was used to assess protein expression on TMA slides. Following antigen retrieval with Diva, quenching of endogenous peroxidase, and rodent block treatments (Biocare), slides were incubated with primary rabbit antibodies [PDI/P4HB (11241-1-AP), PDIA6 (18233-1-AP), ERp57/PDIA3 (15967-1-AP), ERp72/PDIA4 (14712-1-AP), PDIR/PDIA5 (15545-1-AP), AGR3 (11967-1-AP) from Proteintech] for 30-60 minutes. After primary antibody incubation and washing, rabbit polyclonal HRP secondary antibody (Biocare) was applied. Negative controls were obtained by substitution of the primary antibody with Universal Negative reagent (Biocare). Following washing, 3,3-diaminobenzidine (DAB) was applied to visualize all reactions, and slides were counterstained with hematoxylin. The sections were dehydrated through graded alcohols, immersed in xylene, and mounted with coverslips.

TMA slides were scanned using a high throughput pannoramic scanner. Images were then visualized by Case viewer and the percentage of positive staining was calculated by HALO (Indica Labs) software package. Positivity was quantified as the number of positive pixels/mm^2^.

### Ovarian cancer mouse xenograft models

Ten EOC cell lines (Cov318, Caov3, Cov362, HEY, NCI/ADR-RES, OVCAR3, OVCAR5, OVCAR8, SKOV3, and TOV21G) were cultured (See supporting information for cell culture method, histopathology of the tumors from which the cell lines were derived are presented in Supplementary Table 1) and approximately 2-4 x 10^6^ cells of a single cell line were injected subcutaneously into each mouse. We were able to generate xenografts from 7 cell lines (HEY, NCI/ADR-RES, OVCAR3, OVCAR5, OVCAR8, SKOV3, and TOV21G). When tumor size reached approximately 1,000 mm^3^, animals were euthanized and tissue samples were collected. Half of the tumor tissue was preserved for IHC staining and the other half was flash-frozen in liquid nitrogen and stored at -80 °C for Western blotting.

### Preparation of tumor lysate and Western blotting

Tissue samples stored at -80 °C were thawed in RIPA buffer (200 μL to 400 μL) supplemented with proteinase- and phosphatase-inhibitor cocktail (Sigma), then homogenized with an electrical homogenizer followed by short sonication to form a homogeneous tissue lysate. The lysate solution was centrifuged at 18,000 x g for 30 min at 4 °C. Protein concentration was measured with BCA assay (Thermo Fisher). Thirty to forty μg proteins per sample was subjected to SDS-PAGE analysis. Proteins were then electro-transferred to methanol activated immobilon-FL PVDF membranes (EMD Millipore). Membranes were blocked with starting block (Thermo Fisher) for 1 hr at room temperature and incubated with primary antibodies overnight at 4 °C. Dylight 800-conjugated secondary antibodies were used for detection (Thermo Fisher, 1:5000, 5% milk, 1 hour, RT) of fluorescent signal in Odyssey Imaging Systems (LI-COR Biosciences).

### Statistical analysis

Statistical analysis was performed using the unpaired t-test (GraphPad Prism 6.0). Two-tailed *p*-value <0.05 was considered statistically significant. Survival curves were generated using overall survival (OS) data from the UM cohort. For survival curves, we used the conventional Kaplan-Meier method and compared curves using log-rank test, *p*-values < 0.05 were considered to be statistically significant.

## SUPPLEMENTARY MATERIALS FIGURES AND TABLE


