# Supplementary material for: Expression of protein disulfide isomerase family members correlates with tumor progression and patient survival in ovarian cancer
Source: Oncotarget. 2017 Oct 6;8(61):103543–56. doi: 10.18632/oncotarget.21569 (PMC5732749; doi:10.18632/oncotarget.21569)
Supplement: Supplementary file 1 [file oncotarget-08-103543-s001.pdf]

Expression of protein disulfide isomerase family members correlates with tumor progression and patient survival in ovarian cancer

SUPPLEMENTARY MATERIALS

Cell culture

OVCAR-8, OVCAR-5, and OVCAR-3 cells (National Cancer Institute, Developmental Therapeutics Program, Bethesda, MD) were maintained in RPMI-1640 supplemented with 10% heat-inactivated FBS (Gemini-Bioproducts). NCI/ADR-RES cells (National Cancer Institute) were maintained in RPMI-1640 supplemented with 10% heat-inactivated FBS and 5 mmol/L L-glutamine. HEY and Caov-3 cell lines were kindly provided as gifts by Dr. Louis Dubeau (University of Southern California, Keck School of Medicine, Los Angeles, CA). HEY cells were maintained in Dulbecco's Modified Eagle Media (DMEM) supplemented with 10% heat-inactivated FBS and 5 mmol/L L-glutamine. Caov-3 were maintained in MEM supplemented with 10% heat-inactivated FBS. Cells were grown as monolayers at 37ºC in a humidified atmosphere of 5% CO2. COV318, COV362, SKOV3 and TOV21 cells (National Institutes of Health, Bethesda, MD) were maintained in DMEM (COV318 and COV362) and RPMI (SKOV3 and TOV21G) with 10% FBS. To remove adherent cells from the flask for subculture and counting, cells were washed with PBS without calcium or magnesium, incubated with a small volume of 0.25% trypsin–EDTA solution (Mediatech, Inc.) for 5 to 10 minutes, re-suspended with culture medium, and centrifuged. All experiments were carried out using cells in the exponential growth phase. All cells are STR validated. Cells were routinely checked for mycoplasma contamination by using PlasmoTest (InvivoGen).

Isolation of RNA and cDNA preparation

Total RNA was isolated from each cell lines using the RNeasy kit (Qiagen, U.S.A.) according to the manufacturers protocol. The concentration of the RNA preparations were determined by NanoDrop (Thermo Fisher, U.S.A.). The cDNAs was synthesized using 1μg of total RNA in the presence of random primers, dNTPs and reverse transcriptase (cDNA RT kit, Applied biosystem, U.S.A.) following the manufacturer's protocol.

PCR

After a first-strand cDNA synthesis, 5μl of the reaction mix were used to prepare a 20μl PCR mix containing Phusion polymerase (New England Bio Labs, U.S.A) using 2 min at 98˚C, 35 cycle at 98°C for 20S, 52˚C for 30S, 72˚C for 20, 1 at 72°C, 4˚C forever. All primer sequences are presented in Supplementary Figure 8 C. PCR reactions were then subjected to electro-phoresis on 4% agarose gel. Actin used as internal control.

REFERENCES

1. Beaufort CM, Helmijr JC, Piskorz AM, Hoogstraat M, Ruigrok-Ritstier K, Besselink N, Murtaza M, van IJcken WF, Heine AA, Smid M, Koudijs MJ, Brenton JD, Berns EM, Helleman J. Ovarian cancer cell line panel (OCCP): clinical importance of *in vitro* morphological subtypes. PLoS One. 2014;9:e103988.
2. Mitra AK, Davis DA, Tomar S, Roy L, Gurler H, Xie J, Lantvit DD, Cardenas H, Fang F, Liu Y, Loughran E, Yang J, Sharon Stack M, et al. *in vivo* tumor growth of high-grade serous ovarian cancer cell lines. Gynecol Oncol. 2015;138:372-377.
3. Bhatt M, Ivan C, Xie X, Siddik ZH. Drug-dependent functionalization of wild-type and mutant p53 in cisplatin-resistant human ovarian tumor cells. Oncotarget. 2017;8:10905-10918. https://doi.org/10.18632/oncotarget.14228.

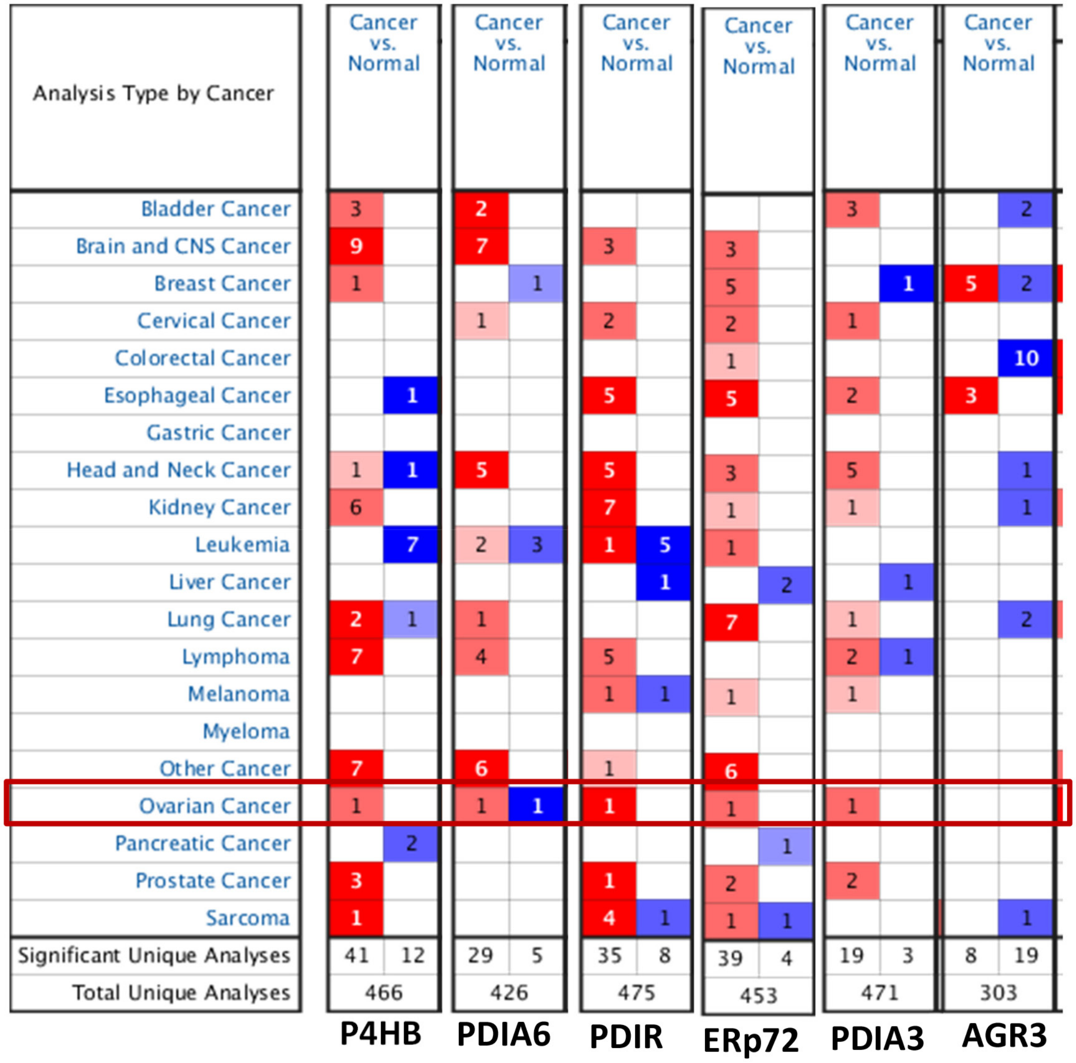

Supplementary Figure 1: PDI and its family proteins are highly expressed in various cancers including ovarian cancer. Figure generated in Oncomine (www.oncomine.com) based on previously published data sets.

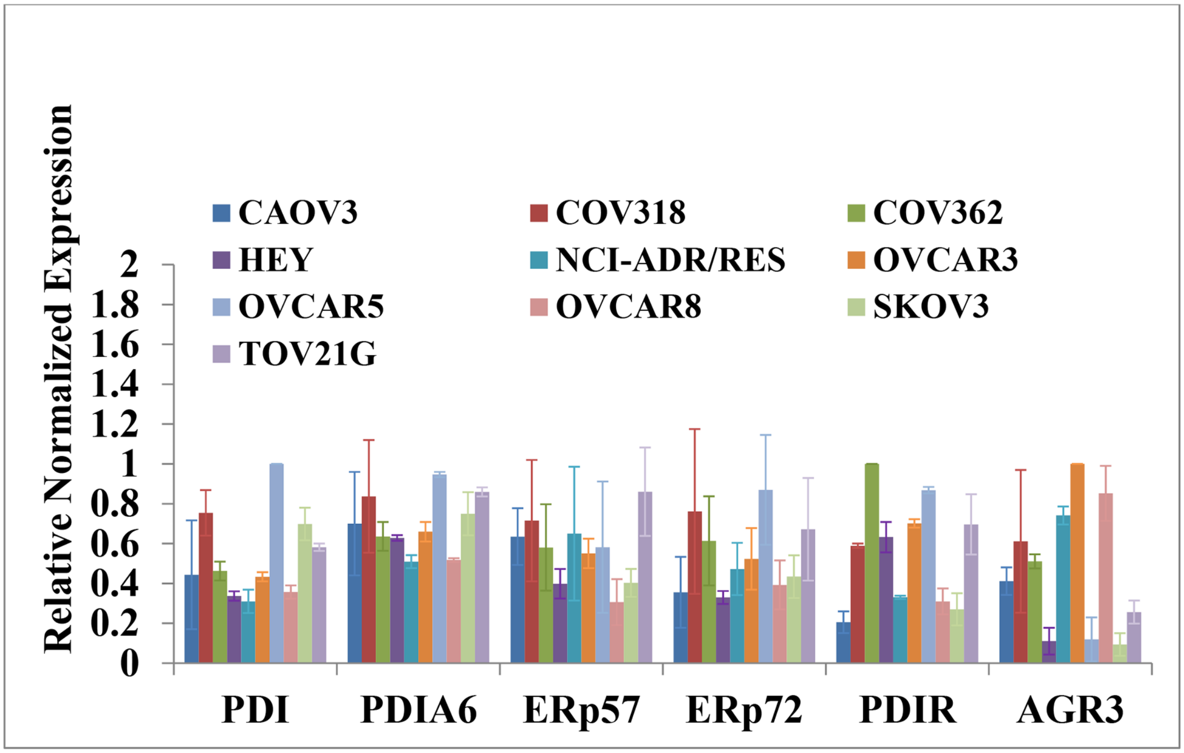

Supplementary Figure 2: Quantification of expression levels of PDIs in a panel of ovarian cancer cell lines. Quantification based on WB data from the 3 independent experiments. Error bars, SD; 1 of 3 representative experiments is shown in Figure 1.

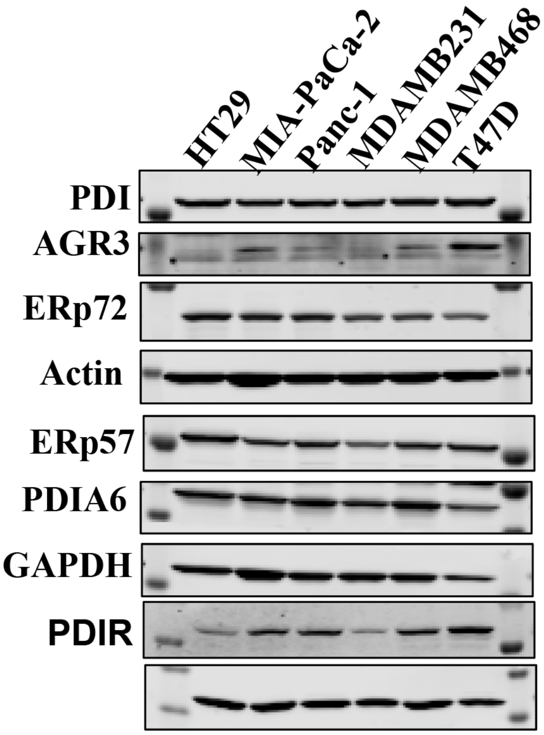

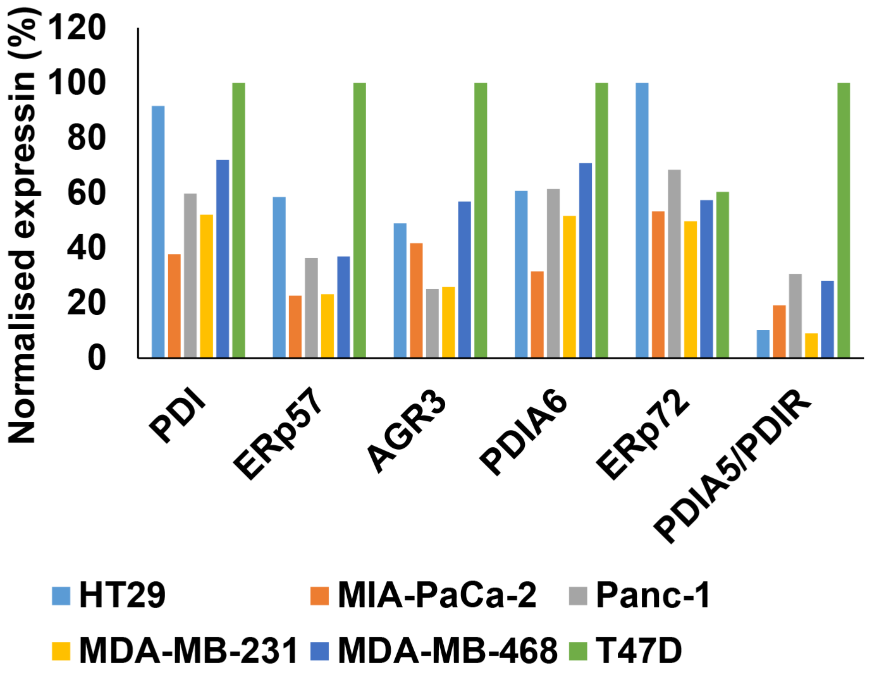

Supplementary Figure 3: Expression levels of PDIs in a panel of non-ovarian cancer cell lines.

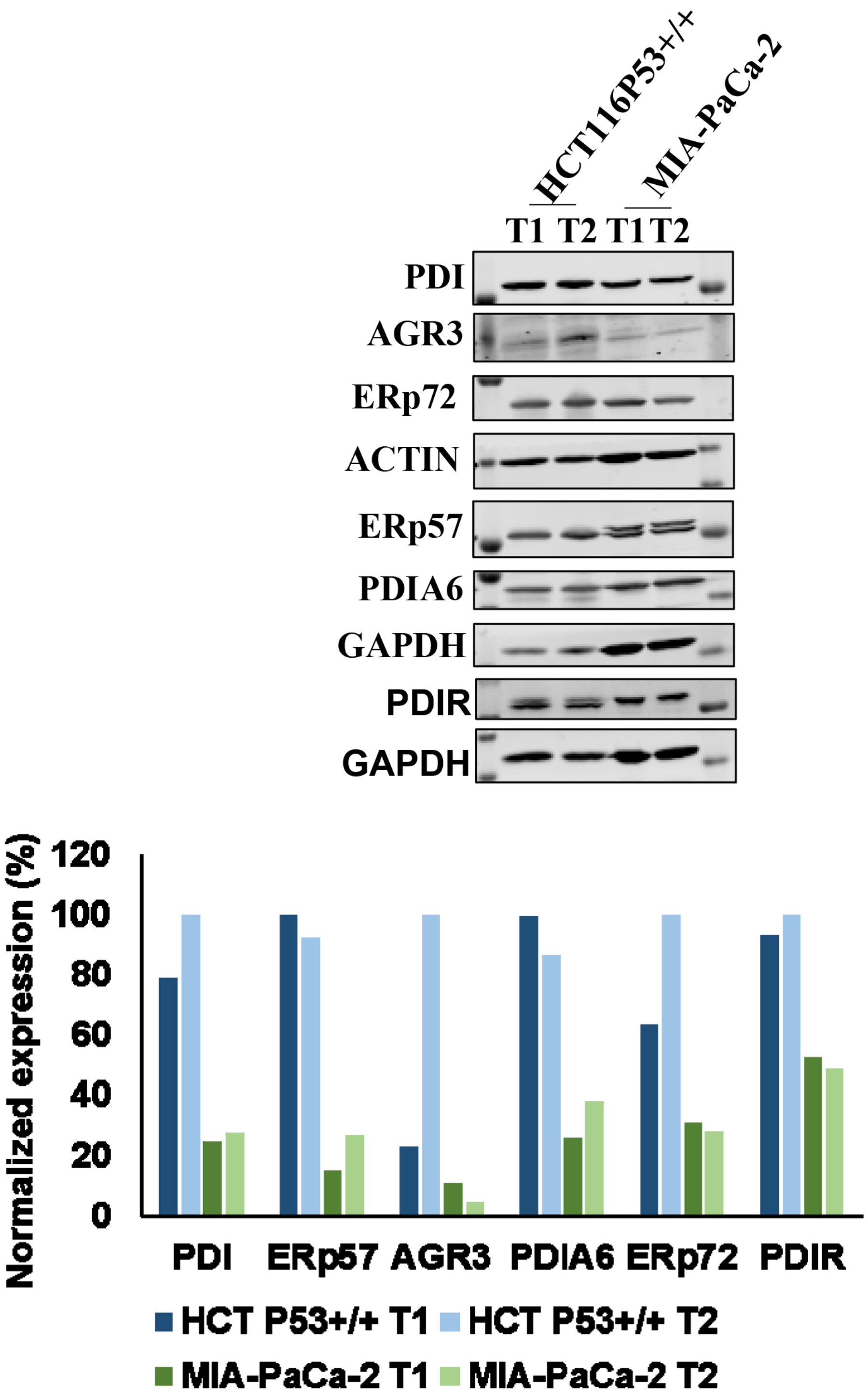

Supplementary Figure 4: Expression levels of PDIs in non-ovarian cancer tissue collected from xenograft.

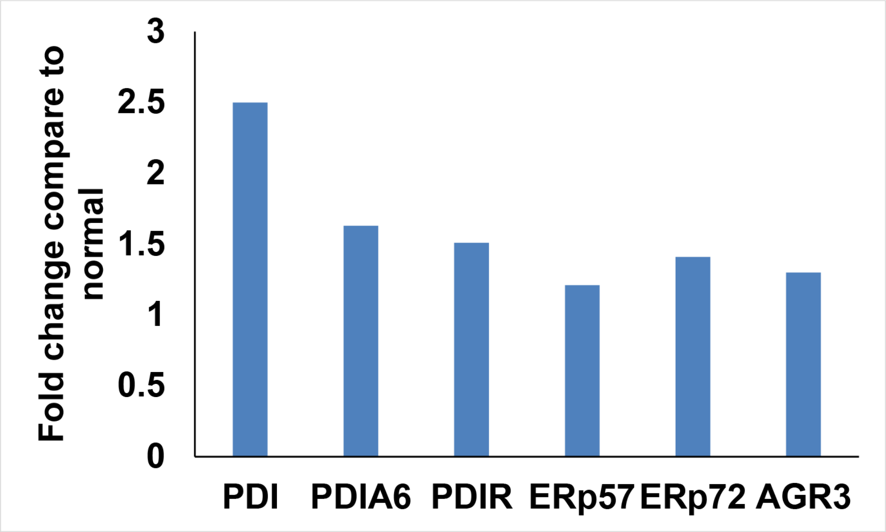

Supplementary Figure 5: Fold change of Expression levels of PDIs compared to normal.

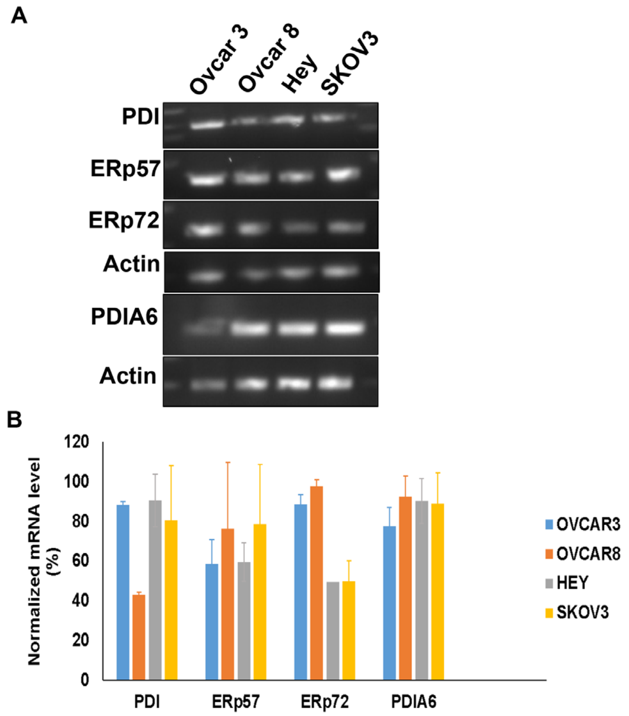

C

Genes	Forward primer	Reverse primer	Size (bp)
PDI	TTG GGA TCA CTT CCA ACA GT	ACA CTC TTG GGC AAG AAC AG	244
ERp57	TCG TCC TTC ACA TCT CAC TAA C	TCC TTG CCC TGT ATC AAA TCT T	159
ERp72	CGC GAG TTT GTC ACT GCT TTC	CGT CCT TCT TGG GGT CCA TC	148
PDIA6	GGAGGTCAGTATGGTGTTCAGGGAT	CTGCCACCTTGGTAATCTTCTGGTC	86
ACTIN	TTG CCG ACA GGA TGC AGA A	GCC GAT CCA CAC GGA GTA CT	101

Supplementary Figure 6: Expression of mRNA levels of PDIs in ovarian cancer cell lines. **(A)** PCR analysis to detect mRNA levels of PDIs in ovarian cancer cell lines (one representative image) **(B)** % of Normalized mRNA levels plotted based on the data collected from at least three experiment. **(C)** Primers information are presented in Table.

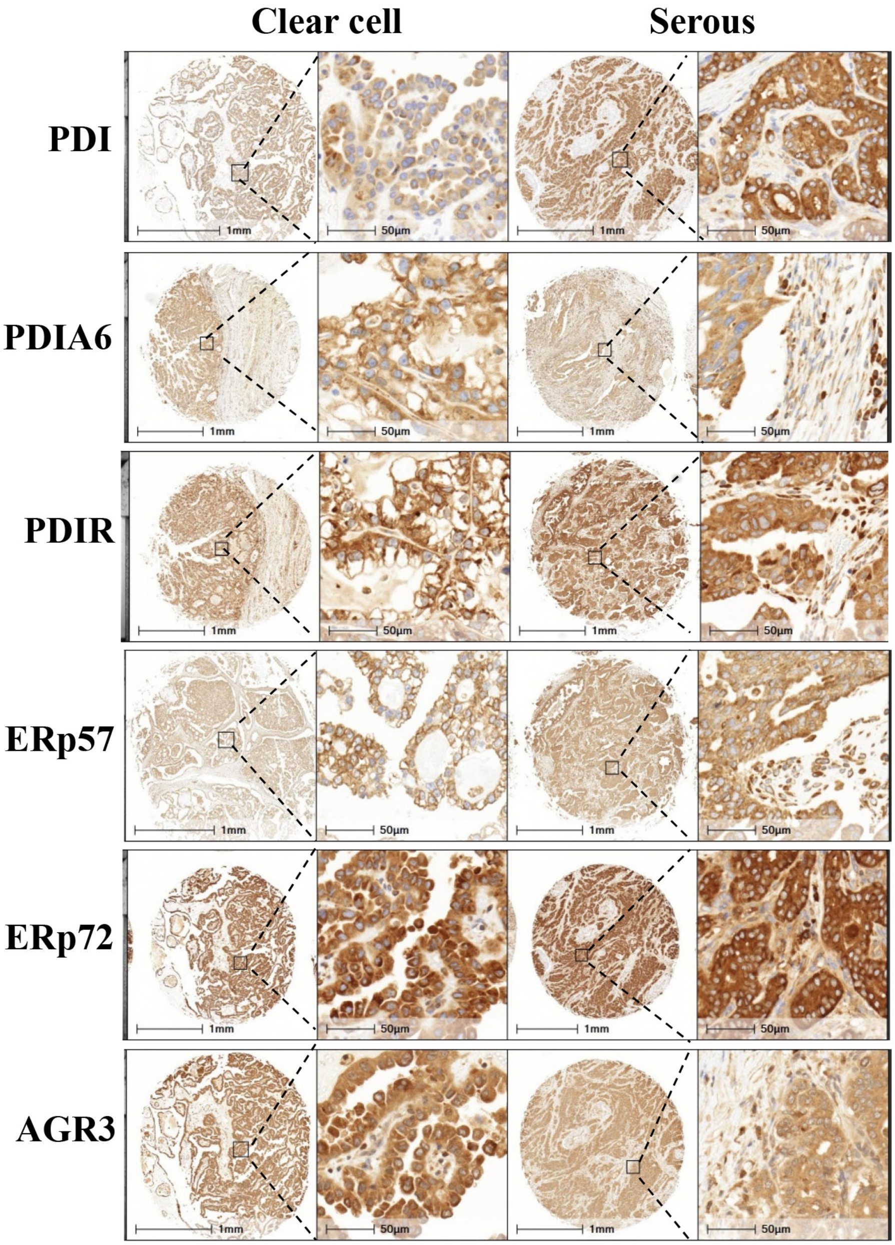

Supplementary Figure 7: Representative staining of PDI, PDI family protein in clear cell and serous ovarian cancer sample.

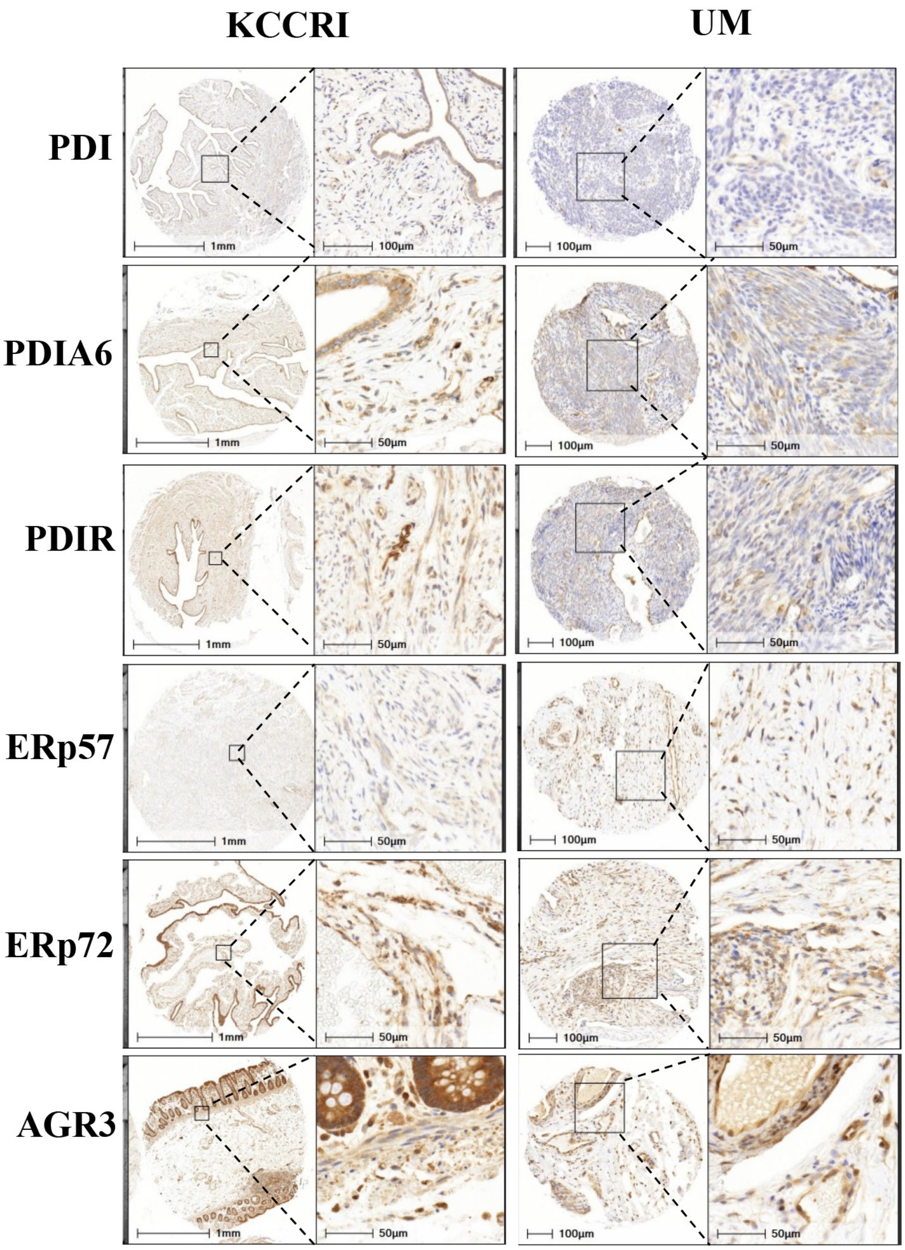

Supplementary Figure 8: Representative staining of PDI, PDI family protein in non-tumor control sample from KCCRI (Japan) and UM (USA) cohort.

Supplementary Table 1: Histology of the ovarian cancer cell lines [Supporting references (1-3)]

Cell line	Histology
CAOV3	Serous
COV318	Serous
COV362	Endometroid
HEY	High grade serous
NCI/ADR RES	High grade serous
OVCAR3	High grade serous
OVCAR5	High grade serous
OVCAR 8	High grade serous
SKOV3	Serous
TOV21G	Clear cell
